# Hybrid Treatment Planning for Chest Wall Irradiation Utilizing Three-Dimensional Conformal Radiotherapy (3DCRT), Intensity-Modulated Radiation Therapy (IMRT), and Volumetric Modulated Arc Therapy (VMAT): A Systematic Review

**DOI:** 10.7759/cureus.59583

**Published:** 2024-05-03

**Authors:** Zainab Alsaihaty, Hanani Abdul Manan, Akmal Sabarudin, Noorazrul Yahya

**Affiliations:** 1 Radiation Therapy, King Fahad Specialist Hospital, Dammam, SAU; 2 Diagnostic Imaging and Radiotherapy, Centre for Diagnostic, Therapeutic and Investigative Sciences, Faculty of Health Sciences, National University of Malaysia, Kuala Lumpur, MYS; 3 Functional Image Processing Laboratory, Department of Radiology, National University of Malaysia, Kuala Lumpur, MYS

**Keywords:** intensity-modulated radiation therapy (imrt), hybrid radiotherapy planning, organs-at-risk, chest wall radiotherapy, vmat radiotherapy

## Abstract

Novel hybrid approaches for chest wall irradiation show promising outcomes regarding target coverage and sparing organs at risk (OARs). In this systematic review, we compared hybrid volumetric modulated arc therapy (H-VMAT) or hybrid intensity-modulated radiotherapy (H-IMRT) techniques with non-hybrid techniques, such as three-dimensional conformal radiation therapy (3DCRT), field-in-field (FIF), intensity-modulated arc therapy (IMRT), and volumetric modulated arc therapy (VMAT), for breast cancer patients with mastectomy. Our focus was the plan quality and dose distribution to the OARs. Using the Preferred Reporting Items for Systematic Reviews and Meta-Analyses (PRISMA) checklist, we performed a systematic review and quality appraisal of primary studies evaluating hybrid therapy to the chest wall and the OARs. An extensive online search of PubMed and Scopus databases was conducted using appropriate keywords. The dose to the OARs (lung, heart, and contralateral breast), planning target volume (PTV), homogeneity index (HI), and conformity index (CI) were extracted. The data were then tabulated and compared for the outcomes between modalities among the studies. Nine studies that met the search criteria were selected to evaluate the PTV coverage and dosimetric results of hybrid and non-hybrid techniques. In terms of 95% PTV coverage, among nine reviewed studies, the largest difference between the two techniques was between VMAT (47.6 Gy) and H-VMAT (48.4 Gy); for the conformity index, the largest difference was noted between 3DCRT (0.58) and H-VMAT (0.79). In both cases, differences were statistically significant (*P* < 0.005). Two studies showed dose homogeneity improvement within the treatment target in H-VMAT (0.15 and 0.07) compared with 3DCRT (0.41 and 0.12), with a *P* value of <0.001. Two studies did not report on the homogeneity index, and three others observed no statistical difference. Regarding OARs, in the comparison of H-VMAT and VMAT, the largest significant change was in the volume receiving 5 Gy (V_5Gy_) of the ipsilateral lung and the V_10Gy_ of the contralateral lung. For the ipsilateral lung, V_5Gy_ was 90.7% with VMAT versus 51.45% with H-VMAT. For the contralateral lung, V_10Gy_ was 54.9% with VMAT versus 50.5% with H-VMAT. In six studies, the mean dose of the contralateral breast was lower in hybrid techniques than in single modalities: VMAT (4.2%, 6.0%, 1.9%, 7.1%, 4.57%) versus H-VMAT (1.4%, 3.4%, 1.8%, 3.5%, 2.34%) and IMRT (9.1%) versus H-IMRT (4.69%). Although most studies did not report on monitor units and treatment time, those that included them showed that hybrids had lower monitor units and shorter treatment times. Hybrid techniques in radiotherapy, such as combining two modalities, can indeed facilitate lower doses to OARs for patients with a high risk of toxicities. Prospective clinical studies are needed to determine the outcomes of breast cancer treated with hybrid techniques.

## Introduction and background

Radiotherapy is a treatment that uses high-energy radiation or radioactive elements to destroy cancer cells while preserving nearby healthy tissues [1]. External beam radiation therapy (EBRT) is a crucial treatment for breast cancer, especially for post-mastectomy patients. In cases where mastectomy has been performed, the indication for radiation therapy is different from breast-conserving treatment, where radiation therapy is an integral part of the treatment. Thus, for patients who are identified to have high-risk features after mastectomy, EBRT is often recommended to reduce the risk of local recurrence by targeting any cancer cells present in the chest wall or nearby lymph nodes. Treatment planning is a crucial component of breast cancer radiation therapy, ensuring that the radiation dose is accurately targeted to the affected area while minimizing exposure to the surrounding healthy tissues.

Radiation treatment planning of breast cancer can be performed using techniques ranging from three-dimensional conformal radiation therapy (3DCRT) and field-in-field (FIF) to more advanced therapies, such as intensity-modulated radiotherapy (IMRT) and volumetric modulated arc therapy (VMAT). In conventional 3DCRT, tangential beams are employed with a simple wedge to avoid low-dose zones in the heart and ipsilateral lung or to create “FIF” or “sub-fields” to the main fields using a multileaf collimator (MLC) [1]. However, these approaches are typically linked to substantially worse homogeneity and compliance and potential hot spots beyond the target volume compared to IMRT and VMAT [2]. Dose homogeneity refers to the uniformity of the radiation dose that is administered throughout the intended area of treatment. However, compliance refers to treatment goals and prescribed dose constraints [3]. Compliance with the treatment plan balances the delivery of radiation dose to achieve therapeutic goals while minimizing the risk of adverse effects on healthy tissues [4].

IMRT is a type of radiation therapy that offers several benefits. It improves dose homogeneity within the target area while preserving critical organs, such as the heart and lungs. However, it has also been found to have potential drawbacks. High monitors (MUs) can increase treatment time and the amount of low-dose radiation exposure, which can potentially increase the risk of developing second malignancies [5].

VMAT has features that help reduce delivery time. It utilizes simultaneous optimization, dynamically adjusting the shape, intensity, and gantry speed of the radiation beam as the machine rotates around the patient. Unlike IMRT, where the gantry stops at various angles to deliver the radiation, VMAT’s gantry continuously rotates. Furthermore, VMAT provides the flexibility to adjust parameters, such as the dose rate and MLC speed, enhancing treatment planning and delivery [6].

IMRT and VMAT both increase the target’s dose conformity at the expense of higher low-dose spread to the contralateral lung and contralateral breast, which may increase the risk of secondary malignancy and other complications [7].

The curvature of the chest wall makes radiation therapy planning for postmastectomy patients more challenging, and the thin target volume along the lung interface makes chest wall irradiation harder than whole breast treatment. Furthermore, depending on the planner’s experience level, sophisticated planning procedures require more extended planning periods [8]. Another problem with breast treatment is target motion brought on by breathing [9]. Therefore, to ensure the accuracy driven by adaptive methods, image-guided radiotherapy (i.e., kilovoltage or megavoltage verification imaging) must be implemented with daily treatment [6]. As it minimizes the margin contributed to the planning target volume (PTV), image-guided radiotherapy has demonstrated an increase in daily treatment accuracy [9]. In addition, deep inspiration breath hold (DIBH) has been used, mainly on the left-sided breast or chest wall, and has considerably reduced the dosage to the heart [10].

In contrast to 3DCRT, IMRT and VMAT are more significantly affected by difficult-to-control breathing patterns [8,11]. Although these state-of-the-art therapies are clinically acceptable, new strategies are needed to successfully lower dosages to the heart and lungs. This need has led to the development of techniques known as hybrid IMRT and VMAT (H-IMRT and H-VMAT, respectively), which combine an open beam or FIF with an inversely planned IMRT or VMAT beam with various weightings.

Mayo et al. [12] introduced a novel technique that integrates static and dynamic fields, known as the hybrid technique, in treating breast cancer patients. This approach was specifically designed to address the crucial concern regarding the protection of OARs, while ensuring optimal treatment plan quality. The strategic integration of these modalities offers potential treatment optimization, particularly in cases related to the breast and the chest wall [13,14]. It is important to acknowledge that each modality has inherent advantages and disadvantages. However, integrating and synergizing these two approaches makes it possible to mitigate the limitations, thus presenting a more refined and enhanced alternative solution.

In this systematic review, we aimed to review and synthesize the comparison between H-VMAT or H-IMRT and non-hybrid techniques for breast cancer, focusing on the plan quality in terms of PTV coverage, homogeneity and conformity indexes, MU, treatment time, and OAR dosages for chest wall cancer patients.

## Review

Material and methods

**Figure 1:**
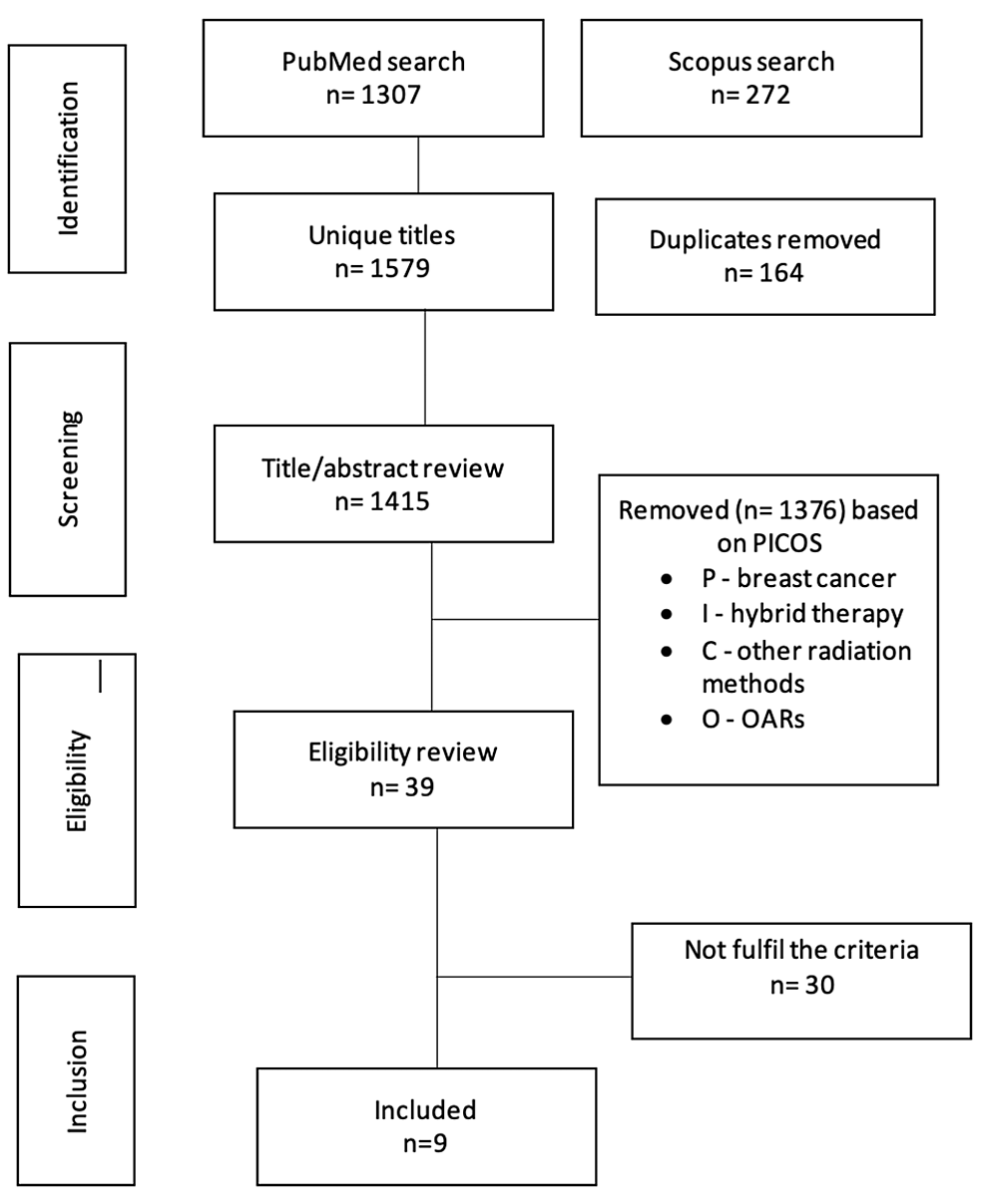
Search strategy via the Preferred Reporting Items for Systematic Reviews and Meta-Analyses (PRISMA) guidelines

The Preferred Reporting Items for Systematic Reviews and Meta-Analyses (PRISMA) checklist was used to guide the design and reporting of this work as a systematic review [15]. The search was conducted in the PubMed and Scopus databases using the population, intervention, control, and outcomes (PICO) framework to establish the search terms (Table 1, Appendix). This type of review is widely used to expand upon the current knowledge base to drive the development of healthcare practice [16-18]. Boolean search phrase combinations were used to guarantee that all pertinent articles were found (Supplementary A). Reference lists were checked for additional studies, and additional manual searches of relevant journals were performed. Following the search, the results were filtered based on the inclusion and exclusion criteria. Duplicates and irrelevant publications were removed from the search by manually filtering the titles and abstracts (Figure 1). The search was further filtered by reading full-text articles to weed out irrelevant publications.

Data Extraction and Synthesis

Authors and year, target, patients, dose prescription, treatment side, breathing setting, hybrid technique, stage, energy, treatment planning system, dose weighting, dose received by 95% (D_95Gy_) of the PTV in Gray(Gy), homogeneity index (HI), conformity index (CI), dose metrics with significant results, planning techniques used, planning or treatment time, MU, the hybrid technique used, type of fields, and the primary outcome of the study were extracted from the selected articles. The dosimetry of all the approaches, including VMAT, IMRT, H-VMAT, H-IMRT, and 3DCRT, was compared between the studies to explore the change in the dose level with different approaches along with their comparable outcomes.

Inclusion Criteria

Studies that involved patients with either left- or right-sided chest wall malignancies and who were candidates for radiation treatment were included. These studies also included the dose parameters of PTV and OARs to facilitate comparison between different treatment techniques.

Exclusion Criteria

Our review excluded studies that involved patients with metastatic disease and those who had previously undergone radiation therapy at the treatment site, as this could increase the risk of radiation-related complications. In addition, studies were excluded if they involved patients treated with other modalities alongside radiotherapy, such as electron and proton therapies.

Results

Study Characteristics

We initially identified 1,579 articles from the PubMed and Scopus searches, and 39 articles were ultimately selected based on the title and abstract. The results were filtered after the search based on the inclusion and exclusion criteria. Articles including whole-breast irradiation were excluded from this review, with the focus directly solely on chest wall irradiation. In addition, studies involving plan robustness were excluded due to their use of phantoms in the study methodology. This exclusion was made because plan robustness in radiotherapy involves quantifying uncertainties and simulating various errors using phantoms, which may not accurately represent the actual uncertainties and variations in patient anatomy and setup. Finally, nine publications were selected and included in the review.

The characteristics of the chosen research articles, which were published between 2018 and 2023, are summarized in Table 1. The nine reviewed articles have various levels of chest wall irradiation with lymph node inclusion. The left side was covered in six reports [19-24]. The chest wall on both sides was studied in three investigations [25-27]. The prescribed doses for the trials that were included ranged from 40.05 to 50.4 Gy in 15 to 25 fractions; the dose weighting for 3DCRT, IMRT, and VMAT ranged from 20% to 80%, and the energy range varied between 6, 10, and 15 MV. In addition, the current review includes reports with different techniques in terms of chest wall side and breathing patterns.

**Table 1:** Characteristics of the included studies Abbreviations: N, number of patients; SCL, supraclavicular; ALN, axillary lymph nodes; IMN, internal mammary node; DIBH, deep inspiration breath hold; G, grade; TPS, treatment planning system; NA, not available; MV, megavolts; H-VMAT, hybrid-volumetric modulated arc therapy; H-IMRT, hybrid-intensity modulated radiation therapy; FIF, field in field.

Reference	N	Nodes included	Dose prescription (no. of fractions)	Treatment side	Breathing setting	Stage or grade	Energy	TPS	Hybrid technique used	Dose weighting	Type of fields used
Balaji et al., 2018 [13]	20	SCL	50.0 Gy (25)	Left	Normal	NA	6 MV	ECLIPSE	H-VMAT (3DCRT+VMAT)	90%/10%, 80%/20%, 70%/30%;	Two coplanar open tangential fields + four coplanar partial arcs
Dumane et al., 2018 [25]	10	SCL, ALN, IMN	50.4 Gy (28)	5 Left + 5 Right	Normal	II–IV	6 MV	ECLIPSE	H-VMAT (3DCRT+VMAT)	NA	3DCRT + two coplanar arcs
Lang et al., 2020 [26]	11	SCL, ALN, IMN	50 Gy (25)	4 Right + 7 Left	DIBH	Locally advanced	6 + 10 MV	ECLIPSE	H-VMAT (3DCRT+VMAT)	80%/20%	Four partial arcs with two additional tangential fields
Doi et al., 2020 [27]	70	SCL, ALN, IMN	50 Gy (25)	35 Left + 35 Right	Normal	G 1, 2, 3	6 MV	ECLIPSE	H- VMAT (3DCRT+VMAT)	NA	Four fields of 3DCRT (two main tangential fields and two anterior-posterior field) + two coplanar arcs
Cilla et al., 2021 [21]	25	SCL, ALN	50.0 Gy (25)	Left	DIBH	G 3	6 MV	PINNACLE	H-IMRT (3CRT+IMRT), H-VMAT (3DCRT+VMAT)	75%/25%	Two open tangential fields and two IMRT fields, two open tangential fields and two partial arcs
Zhang et al., 2022 [23]	32	SCL, ALN, IMN	50 Gy (25)	Left	Normal	G 2, 3	6 MV	ECLIPSE	H-VMAT (3DCRT+VMAT)	70%/30%	Five fields (two tangential fields + two partial arcs + one separate arc)
Sathiyaraj et al., 2022 [24]	15	SCL	50 Gy (25)	Left	NA	NA	6 MV	MONACO	H-VMAT (FIF+VMAT)	70%/30%	FIF for IMRT and two coplanar partial arc for VMAT
Haldar et al., 2023 [22]	10	SCL, ALN	40.5 Gy (15)	Left	NA	NA	6 MV	ECLIPSE	H-IMRT (3DCRT+IMRT)	60%/40%	Two tangential beams and two IMRT fields
Balaji et al., 2023 [20]	25	SCL, IMN	40.5 Gy (15)	Left	DIBH	Locally advanced	6, 10, 15 MV	ECLIPSE	H-IMRT (3DCRT+IMRT), H-VMAT (3DCRT+VMAT) (IMRT+VMAT)	70%/30%	Two tangential (3DCRT) + two tangential IMRT or two partial arcs five IMRT fields + two partial arc

Plan Quality

**Figure 2:**
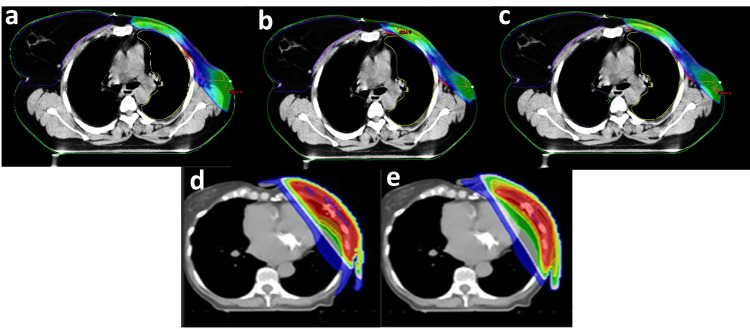
Dose distribution map of a) 3DCRT, b) IMRT, c) H-IMRT, d) VMAT, and e) H-VMAT. a) 3DCRT, b) IMRT, c) H-IMRT; adopted from Haldar et al. (2023) [22]. d) VMAT and e) H-VMAT; adopted from Cilla et al. (2021) [21].

The main findings of the studies comparing the plan quality between hybrid and non-hybrid radiotherapy planning techniques, including MU, treatment time or planning time, HI, and CI, are summarized in Table 2. Various treatment planning strategies and hybrid approaches were used for breast radiotherapy, including 3DCRT, IMRT, VMAT, H-VMAT, and H-IMRT. The dose distribution map for the mentioned techniques is illustrated in Figure 2.

**Table 2:** Significant results of MU, TT, PTV, CI, and HI Abbreviations: AP, automated plan; CI, conformity index; CTV, clinical target volume; Dmin, minimum dose to the PTV; DT, delivery time; FIF, field in field; HI, homogeneity index; MP-VMAT, manual plan-volumetric modulated arc therapy; MU, monitor unit; NA, not available; ns, not statistically significant; PTV(cw), planning target volume for chest wall; PTV, planning target volume; TP, treatment planning; TT, treatment time. ^CTV values.

Authors	Planning techniques used	MU	TT/TP/DT	PTV(cw)/CTV^ P < 0.05	CI P < 0.05	HI P < 0.05	Conclusion
Balaji et al., 2018 [13]	FIF	442	NA	D_95_: 47.4 Gy	0.58	0.12	H-VMAT is better in CI and HI than FIF and significantly better in PTV coverage than VMAT.
	H-VMAT	489	NA	D_95_: 48.4 Gy	0.79	0.07	
	VMAT	524	NA	D_95_: 47.6 Gy	0.87	0.10	
Dumane et al., 2018 [25]	VMAT	NA	NA	V_95_: 97.0%^ns^	NA	NA	The difference in PTV coverage is not statistically significant.
	H-VMAT	NA	NA	V_95_: 96.4%^ns^	NA	NA	
Lang et al., 2020 [26]	VMAT	673^ns^	NA	V_95_: 98.4%	1.15	0.069^ns^	The quality of PTV is maintained compared with VMAT.
	H-VMAT	675^ns^	NA	V_95_: 97.1%	1.21	0.082^ns^	
Doi et al., 2020 [27]	3DCRT	NA	NA	D_min_: 12.3 Gy	2.1	0.41	H-VMAT is better in PTV coverage and HI than 3DCRT.
	H-VMAT	NA	NA	D_min_: 21.6 Gy	1.6	0.15	
Cilla et al., 2021 [21]	H-IMRT	NA	NA	D_95_: 47.1 Gy	NA	51.2	H-IMRT and H-VMAT have the same PTV coverage. H-VMAT is better inhomogeneity than H-IMRT. AP-VMAT is better among all.
	H-VMAT	NA	NA	D_95_: 47.1 Gy	NA	44.8	
	MP-VAMT	NA	NA	D_95_: 48.1 Gy	NA	13.5	
	AP-VMAT	NA	NA	D_95_: 48.5 Gy	NA	12.4	
Zhang et al., 2022 [23]	VMAT	746	DT: 168.6 s	^D_50_: 98.67 Gy	NA	NA	H-VMAT is better in PTV coverage than IMRT. MU delivered by VMAT and H-VMAT is the same but less than three times IMRT.
	IMRT	2098	DT: 365.7 s	^D_50_: 96.97 Gy	NA	NA	
	H- VMAT	742	DT: 169.5 s	^D_50_: 98.31 Gy	NA	NA	
Sathiyaraj et al., 2022 [24]	VMAT	NA	NA	D_95_: 96.9 Gy	0.972^ns^	0.127^ns^	H-VMAT is better in PTV coverage but has no statistical differences in CI and HI.
	H-VMAT	NA	NA	D_95_: 96.2 Gy	0.97^ns^	0.12^ns^	
Haldar et al., 2023 [22]	FIF	323	DT: 32.4 s,	D_95_: 37.10 Gy	0.931	0.111	H-IMRT is lower in MU than IMRT and similar in PTV coverage. H-IMRT is better in CI than FIF. FIF is the lowest in MU, DT, and the minimum in PTV coverage among all. IMRT is better than H-IMRT in HI and CI but not significantly different.
	IMRT	751	DT: 75.0 s	D_95_: 39.32 Gy	0.981^ns^	0.087^ns^	
	H-IMRT	510	DT: 51.0 s	D_95_: 38.39 Gy	0.970^ns^	0.107^ns^	
Balaji et al., 2023 [20]	3DCRT+IMRT	1094	TT: 4.0 min	NA	1.03^ns^	1.08^ns^	All hybrids gave the same homogeneity and conformity. 3DCRT+VMAT gave less time and less MU.
	3DCRT+VMAT	579	TT: 3.2 min	NA	1.03^ns^	1.08^ns^	
	IMRT+VMAT	831	TT: 3.8 min	NA	1.03^ns^	1.09^ns^	

A combination of modalities produced better PTV coverage [19,22,23,27], lower HI [19,27], higher CI [19,22], lower MU [19,22,23], and shorter treatment time [19,22] than relying on a single modality. The hybrid techniques, H-VMAT or H-IMRT, performed better in PTV coverage than single modalities 3DCRT and VMAT. To illustrate, from two studies [19,22], the PTV coverage at 95% of the single modality 3DCRT was 47.6 Gy and 37.10 Gy, better than H-VMAT with 47.4 Gy and 38.39 Gy, respectively. In addition, the PTV coverage was better between VMAT and H-VMAT (47.6 Gy and 48.4 Gy, respectively) [19].

Moreover, HI and CI were also better in hybrid techniques than in IMRT or VMAT [19,22,27]. Dose homogeneity improvement is derived from the calculation of the homogeneity index, where a homogeneity index closer to 1 indicates a more uniform distribution of dose within the target volume, which is considered better. As the homogeneity index increases above one, it indicates a less uniform distribution of dose within the target volume [3]. For example, the HI and CI values for the single modality were 0.41 and 2.1, respectively, while for the hybrid techniques, they were 0.15 and 1.6 [27]. Other studies did not report or did not find a significant change [21,23,25]. H-VMAT and H-IMRT had lower MUs and less treatment time. From two reviewed studies, the MU decreased from 524 and 746 in VMAT to 489 and 742 in H-VMAT [19,23]. Similarly, the MU for IMRT was 751, with a treatment time of 75 s to 510 MU and 51 s in H-IMRT [22].

Organs at Risks (OARs)

The tabulated dosimetric data concerning OARs, focusing on lung (ipsilateral and contralateral), heart, and contralateral breast doses, is summarized in Table 3. Overall, hybrid techniques have demonstrated a reduction in OAR doses compared to VMAT or IMRT but not when compared to 3DCRT. In three separate studies, the volume receiving 5 Gy (V_5Gy_) for the ipsilateral lung was notably higher for VMAT compared to H-VMAT (90.7%, 66.6%, 61.47% vs. 51.45%, 52.4%, 48.84%, respectively). In addition, V_5Gy_ for IMRT was higher than for H-IMRT (36.75% vs. 27.64%, respectively) [19,21,24]. Similarly, V_5Gy_ for the contralateral lung was greater for single-modality approaches compared with the hybrid techniques (VMAT: 35.67% vs. H-VMAT: 0.68% and IMRT: 13.3% vs. H-IMRT: 9.93%) [19,22]. Hybrid techniques were found to be superior in reducing the mean dose (D_mean_) of the contralateral breast in all studies comparing methods: VMAT (ranging from 4.2 Gy to 7.1 Gy) versus H-VMAT (ranging from 1.4 Gy to 3.5 Gy) and IMRT (9.1 Gy) versus H-IMRT (4.69 Gy) [19,21-24,26].

**Table 3:** OAR doses to the lungs, heart, and contralateral breast Abbreviations: AP, automated plan; CB, contralateral breast; CL, contralateral lung; FIF, field in field; H-IMRT, hybrid-intensity modulated radiation therapy; H-VMAT, hybrid volumetric arc therapy; IL, ipsilateral lung; L, left; MP-VMAT, manual plan volumetric arc therapy; ns, not statistically different; OAR, organ at risk; R, right; T-VMAT, tangential volumetric arc therapy; VxGy, volume receiving x Gy.

		OARs (P < 0.05)				
Authors	Techniques	Ipsilateral Lung	Contralateral Lung	Heart	Contralateral Breast	Conclusion
Balaji et al., 2018 [13]	FIF	V_5Gy_: 41.68%	V_5Gy_: 0.00%	D_mean_: 5.41 Gy	D_mean_: 0.54 Gy	H-VMAT is lower in V_5Gy_ lungs and D_mean_ of heart and CB compared with VMAT. FIF is the lowest in all OARs among all techniques.
	H-VMAT	V_5Gy_: 51.45%	V_5Gy_: 0.68%	D_mean_: 6.17 Gy	D_mean_: 1.36 Gy	
	VMAT	V_5Gy_: 90.7%	V_5Gy_: 35.67%	D_mean_: 11.51 Gy	D_mean_: 4.55 Gy	
Dumane et al., 2018 [25]	VMAT	D_mean_: 15.3 Gy	D_mean_: 3.6 Gy	V_5Gy_: 39.0 Gy^ns^	D_mean_: 4.2 Gy	H-VMAT reduced the dose to the CL and CB compared with VMAT. Heart is not statistically significant.
	H-VMAT	D_mean_: 16.4 Gy	D_mean_: 3.2 Gy	V_5Gy_: 40.2 Gy^ns^	D_mean_: 1.4 Gy	
Lang et al., 2020 [26]	VMAT	V_10Gy_: 10.3%	V_10Gy_: 54.9%	D_mean_: 3.7 Gy	D_mean_: 6.0 Gy	H-VMAT is lower in lung (V_10Gy_), heart (D_mean_) and CB (D_mean_) compared with VMAT.
	H-VMAT	V_10Gy_: 6.9%	V_10Gy_: 50.5%	D_mean_: 3.0 Gy	D_mean_: 3.4 Gy	
Doi et al., 2020 [27]	3DCRT,	V_5Gy_: 41.0%	V_5Gy_: 0.0%	D_mean_: 11.8 Gy^ns^	NA	V_5Gy_ of IL and CL are higher in H-VMAT compared with 3DCRT. Heart (D_mean_) is not statistically significant.
	H-VMAT	V_5Gy_: 47.5%	V_5Gy_: 28.0%	D_mean_: 12.0 Gy^ns^	NA	
Cilla et al., 2021 [21]	H-IMRT	V_5Gy_: 49.7%	NA	V_5Gy_: 5.3%	D_mean_: 1.3 Gy	H-IMRT is lower in lungs, heart and CB when compared with H-VMAT. Both I-IMRT and H-VMAT is lower in lungs, heart, and CB compared with manual plan VMAT. AP-VMAT is lower in heart.
	H-VMAT	V_5Gy_: 52.4%	NA	V_5Gy_: 6.1%	D_mean_: 1.8 Gy	
	MP-VMAT	V_5Gy_: 66.6%	NA	V_5Gy_: 6.6%	D_mean_: 1.9 Gy	
	AP- VMAT	V_5Gy_: 55.7%	NA	V_5Gy_: 4.1%	D_mean_: 2.3 Gy	
Zhang et al., 2022 [23]	T-VMAT	V_20Gy_: 22.2%	V_20Gy_: 2.77%	V_30Gy_: 2.13%	D_mean_: 7.1 Gy	Only CB is the lowest in H-VMAT among all.
	IMRT	V_20Gy_: 29.6%	V_20Gy_: 0.17%	V_30Gy_: 7.15%	D_mean_: 3.6 Gy	
	H- VMAT	V_20Gy_: 28.81%	V_20Gy_: 0.18%	V_30Gy_: 8.53%	D_mean_: 3.5 Gy	
Sathiyaraj et al., 2022 [24]	H-VMAT	V_5Gy_: 48.84%	D_mean_: 2.19 Gy	V_30Gy_: 3.27%	D_mean_: 2.34 Gy	H-VMAT is lower in IL (V_5Gy_), D_mean_ of CL and CB. Heart is lower in VMAT.
	VMAT	V_5Gy_: 61.47%	D_mean_: 3.9 Gy	V_30Gy_: 2.94%	D_mean_: 4.57 Gy	
Haldar et al., 2023 [22]	FIF	V_5GY_: 29.96%	D_mean_: 9.7 Gy	V_10Gy_: 11.94%	Dmax: 3.67 Gy	H-IMRT is lower in V_5Gy_ lungs and V_10Gy_ heart than IMRT. CB is the lowest in FIF among all.
	IMRT	V_5GY_: 36.75%	D_mean_: 13.3 Gy	V_10Gy_: 14.22%	Dmax: 9.19 Gy	
	H-IMRT	V_5GY_: 27.64%	D_mean_: 9.93 Gy	V_10Gy_: 10.55%	Dmax: 4.69 Gy	
Balaji et al., 2023 [20]	3DCRT+IMRT,	V_5Gy_: 56.5%	V_5Gy_: 1.6%	V_5Gy_: 30.4%	V_5Gy_: 6.0%	3DCRT+IMRT and 3DCRT+VMAT are lower in IL and heart. IMRT+VMAT is lower in CB.
	3DCRT+VMAT,	V_5Gy_: 57.3%	V_5Gy_: 0.6%	V_5Gy_: 30.6%	V_5Gy_: 4.4%	
	IMRT+VMAT	V_5Gy_: 60.4%	V_5Gy_: 0.6%	V_5Gy_: 41.5%	V_5Gy_: 3.5%	

Discussion

This article is the first systematic review to examine hybrid planning techniques for chest wall irradiation and compare them with other hybrid or non-hybrid techniques. We compared the effectiveness of H-VMAT or H-IMRT and non-hybrid techniques (3DCRT, FIF, IMRT, and VMAT), emphasizing the quality of the treatment plan and the dose to the OARs. Although reports on PTV coverage, CI, HI, and doses to OARs are conflicting, hybrid techniques generally provide better PTV coverage than single modality techniques (3DCRT, IMRT, and VMAT) and result in lower doses to OARs, such as the lungs, heart, and contralateral breast.

In the majority of institutions around the world, 3DCRT is the most popular radiation treatment planning technique for breast cancer [1]. The technique is used in many reviewed studies along with post-mastectomy hybrid planning radiotherapy [19-21,23-27]. In those investigations, 3DCRT was used either with two open tangential fields or two tangential FIF [19,22,27]. These tangential beams avoid exposing the ipsilateral lung and heart to low-dose radiation, but they have poor conformance and homogeneity [2]. Due to the target’s conformance, IMRT and VMAT are now frequently recommended approaches over 3DCRT for pelvic malignancies, prostate cancer, and head and neck tumors [6]. With regard to postmastectomy breast radiation, there is considerable discussion on using these methods. The importance of IMRT alone or in combination with other approaches for chest wall irradiation was demonstrated in some studies [20,22]. According to reports, IMRT doubles the likelihood of developing subsequent cancer compared with 3DCRT [28,29]. As a result, several studies used VMAT alone or in combination with other planning modalities [19,20,23-27], possibly due to shorter treatment times and MU, which reduces the total exposure and potentially reduces the risk of radiation-induced secondary tumors [6].

Some institutions are conducting ongoing trials of hybrid techniques, while others are already in operation [21]. This review shows that there are different suggested hybrid techniques for chest wall irradiation, which are a combination of either 3DCRT (OF or FIF) + VMAT [19,20,22-27] or 3DCRT (OF or FIF) + IMRT [20,22,24]; three studies have used IMRT+VMAT [20,23,24].

It is evident from some studies that the H-VMAT (3DCRT+VMAT or FIF+VMAT or IMRT+VMAT) is superior in providing conformity, uniformity, and dose reduction to OARs compared with VMAT alone or FIF [19-24,26]. However, only two studies claimed that the PTV coverage maintained the same quality as VMAT alone [25,27]. One study compared H-IMRT (3DCRT+IMRT) with H-VMAT (3DCRT +VMAT) and H-VMAT (IMRT +VMAT) and concluded that H-VMAT (3DCRT +VMAT) is superior [20]. Another study compared H-IMRT (3DCRT+IMRT) with IMRT alone and 3DCRT and stated that H-IMRT is the best option for homogeneity and dose distributions [22].

IMRT for breast cancer is limited by uncertainties in patient setup and respiratory motion causing unexpected dose deviations [30]. The H-IMRT plan may eliminate the geometrical errors associated with IMRT by combining two opposed tangential open beams with IMRT beams. In terms of robustness against uncertainty and plan quality, H-IMRT outperformed the non-hybrid IMRT [30]. Many challenges in treating the chest wall area must be considered in determining the optimal planning technique. The physical characteristics of patients can vary between individuals, in terms of the size and volume of the PTV, chest wall separation, heart volume and position, and lung volume [19]. In addition, age, breathing motion, and daily setup reproducibility further contribute to the challenges encountered in planning cases involving the chest wall [2].

Beam weighting refers to the process of assigning different weights to the technique involved. It serves as an additional selection criterion after identifying the optimal technique. Balaji et al. [19] demonstrated that H-VMAT with beam weighting (80-90% of 3DCRT and 10-20% of VMAT) is the optimal choice based on its correlation with mean dose, V_5Gy_, and V_20Gy_ of the heart and lung, as well as the lower incidence of secondary cancers resulting from low-dose irradiation. Furthermore, several other studies [23,25,26] have provided support for the superiority of the beam-weighting methods described by Balaji et al. [19].

This review highlights various suggested techniques for chest wall radiation. Utilizing newer techniques, such as IMRT/VMAT, which aim to achieve good PTV coverage, may increase low-dose irradiation to OARs compared with traditional 3DCRT plans. In addition, when the internal mammary chain is involved in the treatment of the chest wall area, it poses an increased risk to the heart. However, IMRT and VMAT are preferred over other techniques due to their ability to create concave dose distributions for the internal mammary chain [31].

Studies have reported that the risk of coronary artery disease is reduced with doses ≤30 Gy, indicating that V_25Gy_ should be less than 10% [32]. Furthermore, it has been observed that the incidence of coronary events increases by 7.4 per 1 Gy increase in the mean heart dose [33]. In most studies, hybrid techniques (H-VMAT, H-IMRT) had lower heart doses than VMAT or IMRT [13-18,20], but some studies reported no statistically significant difference [25,27]. However, these latter studies included both breast and chest wall patients, which could affect the final result. One study performed an automated plan of VMAT and found statistical differences in the doses to the heart compared with the manual VMAT plan, H-IMRT, and H-VMAT [21]. Radiation-induced pneumonitis can be caused by radiation doses to the lungs on the same side [34]. Achieving optimal lung health requires exceptional care in optimizing both sides of the lungs. With free-breathing or DIBH settings, H-VMAT performs better [19-21,23,24,26]. Two studies of breast mastectomy showed that in a comparison between H-VMAT and H-IMRT, H-IMRT was better in V_5Gy_ [20,21]. Similarly, in a comparison of IMRT with H-IMRT, the V_5Gy_ and V_10Gy_ of ipsilateral lung were smaller in H-IMRT than in IMRT and FIF [22]. The parameters V_5Gy_, V_10Gy_, and V_20Gy_ of the ipsilateral lung are known as predictors of radiation-induced pneumonitis, but the most effective predictor among these parameters has been debated in several studies [34]. H-VMAT was shown to be considerably reduced in D_mean_, V_5Gy_, and V_20Gy_ in several investigations [19,23-27].

In addition, it is essential to note that advanced techniques carry a potential risk to the contralateral breast. Ignoring this factor during the planning process may result in radiation-induced carcinogenesis [35]. The D_mean_ of the contralateral breast is less with the hybrid technique (H-VMAT; either 3DCRT+VMAT or IMRT+VMAT) than with the non-hybrid technique (VMAT) [19,23-26]. Only one study reported no radiation to the contralateral breast when using a hybrid technique as no VMAT was used over the chest area [27]. The D_mean_ of H-IMRT was reported to be better than that of H-VMAT [21]. Similarly, in comparison with IMRT [22], both H-VMAT and H-IMRT had lower doses to the contralateral breast in comparison with VMAT alone [21].

It has been reported that the more MU delivered to the target, the higher the dose to normal tissues and the greater the incidence of radiation-induced malignancies [6]. Although four of the studies reviewed did not report details on MU, H-VMAT or H-IMRT had a smaller MU than the other techniques [19,20,22,23]. In terms of treatment time, H-VMAT was found to have comparatively less time than IMRT or VMAT [22,36]. The shorter time brought more comfort to the patients, especially in DIBH settings, increasing reproducibility and reducing setup errors.

The dosimetric parameters for both PTV and OARs varied between studies, and the field involvement of hybrid techniques also differed. Some studies only addressed certain combinations of modalities for chest walls and regional nodes. Furthermore, many studies did not report on crucial factors, such as CI, HI, and MU alongside treatment time. Due to these inconsistencies, it is challenging to come to a definitive conclusion.

Maintaining a consistent approach to dose prescription, target volume, and dose constraints is essential when reporting on breast radiotherapy treatment plans. In cases in which both sides of the breast are treated, it is vital to evaluate each side individually or to assess the heart doses if possible. Determining the breathing pattern is highly recommended for the precise evaluation of OAR structures. In addition, a larger population is needed to achieve more accurate and reliable outcomes.

## Conclusions

The hybrid planning technique is potentially valuable in the treatment of postmastectomy breast irradiation. However, few comparative dosimetric studies of chest wall volumes and nodal volumes have been addressed in existing research. Further investigation of combining availability modalities is needed with the use of automated plans for chest wall patients. Since the studies did not use flattening filter-free beams for chest wall irradiation, further investigation comparing 3DCRT+IMRT, 3DCRT+VMAT, and IMRT+VMAT is needed. In addition, no long-term clinical outcome studies have been conducted in patients treated with hybrid procedures, necessitating in-depth prospective research to investigate the benefits of the hybrid approaches.

## Appendices

Online search of PubMed and Scopus databases

( TITLE-ABS-KEY ( breast  AND  cancer )  OR  TITLE-ABS-KEY ( breast  AND  carcinoma ) )  AND  ( TITLE-ABS-KEY ( radiotherapy )  OR  TITLE-ABS-KEY ( radiation  AND  therapy ) )  AND  ( TITLE-ABS-KEY ( hybrid  AND  plan )  OR  TITLE-ABS-KEY ( integrated  AND  plan )  OR  TITLE-ABS-KEY ( combined  AND  plan ) )  AND  ( TITLE-ABS-KEY ( oars )  OR  TITLE-ABS-KEY ( organs  AND at  AND risk )  OR  TITLE-ABS-KEY ( lung )  OR  TITLE-ABS-KEY ( heart )  OR  TITLE-ABS-KEY ( surrounding  AND organs ) ) 

Scopus: 272 documents

Search: ((((breast cancer) OR (breast carcinoma)) AND ((radiotherapy) OR (radiation therapy))) AND (((hybrid plan) OR (Integrated plan)) OR (Combined technique))) AND (((((OARs) OR (Organ at risks)) OR (Surrounding organs)) OR (heart)) OR (lung))

PubMed: 1,307 documents
